# Structure Prediction of Rare Earth Doped BaO and MgO Containing Aluminosilicate Glasses–the Model Case of Gd_2_O_3_

**DOI:** 10.3390/ma11101790

**Published:** 2018-09-20

**Authors:** Mohamed Zekri, Andreas Erlebach, Andreas Herrmann, Kamel Damak, Christian Rüssel, Marek Sierka, Ramzi Maâlej

**Affiliations:** 1 Georesources Materials Environment and Global Changes Laboratory (GEOGLOB), Faculty of Sciences of Sfax, Sfax University, 3018 Sfax, Tunisia; mohamed.zekri.etud@fss.usf.tn (M.Z.); Kamel.Damak@fss.rnu.tn (K.D.); ramzi.maalej@fss.usf.tn (R.M.); 2 Otto-Schott-Institute of Materials Research, Jena University, 07743 Jena, Germany; andreas.herrmann@uni-jena.de (A.H.); ccr@uni-jena.de (C.R.); marek.sierka@uni-jena.de (M.S.)

**Keywords:** glass, atomistic simulations, rare earth, aluminosilicates

## Abstract

The medium-range atomic structure of magnesium and barium aluminosilicate glasses doped with Gd_2_O_3_ as a model rare earth oxide is elucidated using molecular dynamics simulations. Our structure models rationalize the strong dependence of the luminescence properties of the glasses on their chemical composition. The simulation procedure used samples’ atomic configurations, the so-called inherent structures, characterizing configurations of the liquid state slightly above the glass transition temperature. This yields medium-range atomic structures of network former and modifier ions in good agreement with structure predictions using standard simulated annealing procedures. However, the generation of a large set of inherent structures allows a statistical sampling of the medium-range order of Gd^3+^ ions with less computational effort compared to the simulated annealing approach. It is found that the number of Si-bound non-bridging oxygen in the vicinity of Gd^3+^ considerably increases with growing ionic radius and concentration of network-modifier ions. In addition, structure predictions indicate a low driving force for clustering of Gd^3+^, yet no precise correlation between the atomic structure and luminescence lifetimes can be conclusively established. However, the structure models provided in this study can serve as a starting point for future quantum mechanical simulations to shed a light on the relation between the atomic structure and optical properties of rare earth doped aluminosilicate glasses.

## 1. Introduction

Aluminosilicate glasses have widespread use in many different technological fields, e.g., as optical devices, as high strength display glasses, or as precursors for low expansion or bio-glass ceramics. Display glasses are mostly based on sodium aluminosilicate glass compositions [1,2]. An ion exchange process, during which near-surface Na^+^ ions are exchanged against much larger K^+^ ions is utilized to achieve high tensile strengths of more than 800 MPa [3] since this process is carried out at temperatures much below the glass transition temperature. Therefore, mechanical stresses cannot relax, and compressive stresses are formed near the surface. Other applications of aluminosilicate glasses are precursors for glass ceramics, especially for low thermal expansion glass ceramics. These are usually based on the Li_2_O/Al_2_O_3_/SiO_2_ system and enable the precipitation of negative thermal expansion crystal phases such as β-quartz [4]. High strength glass ceramics can also be prepared based on the system MgO/Al_2_O_3_/SiO_2_ [5,6]. Here, recently tensile strengths of more than one GPa have been reported [7]. Most of the currently prepared aluminosilicate glasses are alkaline or alkaline earth aluminosilicate glasses. 

In the past few years, aluminosilicate glasses doped with rare earth ions have also been proposed as laser materials [8]. They possess excellent thermo-mechanical properties, such as high strength, high hardness, high toughness, and a low coefficient of thermal expansion (CTE) [9] and, therefore, also a much higher laser damage threshold than conventionally used laser glass compositions such as phosphate and fluoride phosphate glasses [10]. However, exploitation of these beneficial mechanical properties for the design of novel laser materials also requires tailored luminescence properties. Recently, several studies on rare earth doped aluminosilicate glasses were published. As dopants, ytterbium, samarium, europium, and other ions were used [11,12,13,14]. It was shown that the base composition of glass has a strong impact on the luminescence properties of the dopant rare earth ions, e.g., the spectral shape of their absorption and emission spectra as well as their luminescence lifetimes. In general, glass compositions with network-modifier ions of low electric field strength (large ion radius and relatively low electric charge, as e.g., Ba^2+^ or K^+^) provide a notably increased splitting of the emission and absorption peaks of the dopant rare earth ions. Additionally, a lifetime increasing effect in peralkaline glass compositions with low field strength network-modifier ions was found. These relations were observed for several different rare earth ions such as Sm^3+^ [12], Eu^3+^ [11,12], and Tb^3+^ [14]. However, up to now, this behavior could not be rationalized making the targeted design of aluminosilicate glasses for laser applications a cumbersome process. Hence, it is an important task to investigate the atomic structure of these glasses, and especially the local surrounding of the dopant rare earth ions, in order to determine the fundamental relationships between the atomic structure and luminescence properties. 

The structure of aluminosilicate glasses has been mainly studied using Nuclear Magnetic Resonance (NMR) and Raman spectroscopy. The glasses can roughly be categorized according to their network-modifier oxide (NMO) to aluminum oxide ratio NMO/Al_2_O_3_. In glasses with a NMO/Al_2_O_3_ ratio > 1, aluminum is mainly incorporated as [AlO_4_]^−^ tetrahedra whose negative charges are balanced by network-modifying cations. The cations that are not needed for charge compensation give rise to the formation of non-bridging oxygen sites. In glasses with an NMO/Al_2_O_3_ ratio < 1, a part of the aluminum is incorporated as [AlO_4_]^−^ tetrahedra whose charge is balanced by network-modifying cations, and additionally aluminum is incorporated in five- or six-fold coordination or as triclusters [15]. In such glasses, non-bridging oxygen sites should not occur. By contrast, due to the lack of appropriate spectroscopic methods, little is known about structural effects of the incorporation of rare earth ions.

Due to the limitation of experimental methods, the full structural information at an atomic level can only be obtained with the aid of simulations. In particular, molecular dynamics (MD) simulations proved essential to aid the resolution of the atomic structure of glasses [16,17]. The simulation procedure frequently used for glass structure predictions—the simulated annealing—employs an equilibration at high temperatures (far above the glass transition temperature, *T*_g_), followed by a stepwise temperature reduction below *T*_g_ to locate low energy structures. For reliable predictions of the medium-range atomic structure of amorphous materials, the statistical sampling of the potential energy surface (PES) employing structure models containing about 10^2^–10^4^ atoms, is essential [18]. In case of glass compositions with low dopant concentrations of about 1 mol%, the size of the structure models has to be substantially larger (>10^4^ atoms [16]) to achieve a sufficient statistical sampling of the medium-range atomic order of the dopant ions. However, this results in a considerable increase of the computational effort for structure predictions of such glasses. 

Instead of locating one low energy structure using one very large structure model (>10^4^ atoms), a potential alternative could be the generation of a large set of atomic configurations that emerge with a certain probability during the glass transition but contain fewer atoms in the unit cell. By performing MD simulations of the liquid state at *T* > *T*_g_, the ions have sufficient kinetic energy to overcome barriers of the PES and to diffuse through the glass structure. Structure optimizations of such MD trajectories yield so-called inherent structures (IS), a concept initially proposed by Stillinger and Weber [19,20], along with the probability distribution that a configuration extracted from the equilibrated liquid state is characterized by the corresponding IS. Since each of those inherent structures characterizes a particular basin of the PES, the 3*N*-dimensional configuration space and the partition function can be separated into non-overlapping basins [20]. Therefore, simulations using this concept are capable to predict not only the atomic structures, but also thermodynamic quantities of glass-forming liquids at temperatures close to *T*_g_ [21,22]. However, to the best of our knowledge, such simulation procedures have not yet been applied for structure predictions of multicomponent glasses containing a dopant with very low molar concentration. 

In this article, structure predictions using MD simulations of magnesium and barium aluminosilicate glasses doped with Gd_2_O_3_ as a model rare earth oxide are presented. The two different glass systems have been chosen because of the large difference in the size and electric field strength of the Mg^2+^ and Ba^2+^ ions, their different effect on the luminescence properties of the doped rare earth ions and their presumably different structural role in the aluminosilicate glass network. In addition, the local surrounding of the Gd^3+^ ions within these glasses is analyzed in detail.

## 2. Computational Details

Molecular dynamics (MD) simulations employed the Large-Scale Atomic/Molecular Massively Parallel Simulator (LAMMPS, Sandia National Laboratories, Albuquerque (New Mexico), USA) [23] along with the empirically parameterized interatomic potential functions of Pedone et al. [24]. This parameterization proved to accurately predict the atomic structure, transport, and mechanical properties of various oxides, silica-based glasses, as well as rare earth doped glasses [25,26,27,28]. Computation of short-range interactions used a cutoff of 15 Å, and for summation of long-range Coulomb interactions the particle-particle-particle-mesh method [29] was applied. Integration of the equations of motion used the velocity Verlet algorithm [30] with a timestep of 1 fs.

Structure predictions were performed using 3D periodic glass structure models, the chemical compositions of which are summarized in Table 1. Due to their very different cation radii, MgO and BaO were chosen as network-modifier oxides (NMO). For both NMO, the chemical compositions of the undoped glass models are *x* NMO·(40 − *x*)Al_2_O_3_·60 SiO_2_ with *x* = 20 mol% (metaluminous glass composition: **20Mg**, **20Ba**) and *x* = 30 mol% (peralkaline glass composition: **30Mg**, **30Ba**). Since the same dependence of the luminescence properties on the glass composition was observed in experiments irrespective of the rare earth ion used (Sm^3+^, Eu^3+^, Tb^3+^, Dy^3+^) [11,12,14], Gd^3+^ was chosen as model rare earth ion because of its similar ionic radius [31] and the available interatomic potential parameters. All structure models contain approximately 1 mol% Gd_2_O_3_.

Glass structure prediction employed the following computational procedure, denoted as inherent structure sampling (ISS). First, initial structure models were generated by randomly placing ions in cubic unit cells (using an in-house script) with volumes corresponding to the experimentally observed mass densities [12]. Next, the initial structures were geometrically optimized and equilibrated at *T* = 3000 K for 6.5 ns using the canonical (NVT, constant particle number N, volume V and temperature T) ensemble along with the Nosé-Hoover thermostat [32,33]. During the last 6 ns, geometry optimizations under constant (zero) pressure conditions were applied to structures taken from the MD trajectory every 2 ps yielding 3000 inherent structures (IS), each with a potential energy per atom *e*_IS_. The variation of cell parameters during the structure optimizations allowed anisotropic unit cell deformations. The obtained distribution of the IS potential energies *P*(*e*_IS_,*T*) corresponds to the probability distribution that a configuration in the liquid state at temperature *T* is related to an IS with energy *e*_IS_ [21,22]. In general, *P*(*e*_IS_,*T*) is strongly temperature dependent showing a shift to lower *e*_IS_ with decreasing *T*, when the sampling is performed at temperatures *T* slightly above *T*_g_ [34]. However, assuming that the structural changes of the average medium-range order of the glass structure between *T* and *T*_g_ are relatively small, *P*(*e*_IS_,*T*) can be used to calculate a mean IS that resembles the lower energy structures at *T*_g_ and, consequently, temperatures below. Therefore, the ensemble average 〈*X*〉 of properties such as pair distribution functions of the macroscopic, amorphous state is approximately calculated from the probability *P_i_* of *n* inherent structures *i* and their related properties *X_i_* using the weighted average:

(1) $$\langle X\rangle =\overset{n}{\underset{i=1}{\sum }}P_{i}X_{i}.$$

In order to test the accuracy of the ISS, the procedure was repeated at different temperatures (2500 and 5000 K) in the case of **30Ba**. In addition, to verify the reliability of the ISS for glass structure predictions, a simulated annealing (ANN) procedure was applied for **30Ba**. Due to the similar unit cell compositions, see Table 1, it is assumed that the complexity of the PES is approximately the same for every structural model. Consequently, it is expected that the ANN for **30Ba** is a sufficient test case. The starting point for the ANN was a randomly generated initial structure model using the unit cell composition Gd_32_Ba_660_Al_440_Si_1320_O_4008_ (6460 atoms). The geometrically optimized initial structure was equilibrated at 5000 K using the canonical (NVT) ensemble. After an equilibration time of 300 and 350 ps, respectively, two low-energy structures (ANN1, ANN2) were obtained by two independent MD simulations using a linear temperature reduction down to 300 K with a cooling rate of 1 K/ps (duration 4.7 ns). The temperature was adjusted every timestep by applying velocity scaling. Finally, both structures were optimized under constant (zero) pressure conditions. 

## 3. Results and Discussion

### 3.1. Comparison of Simulated Annealing (ANN) and Inherent Structure Sampling (ISS)

**Figure 1:**
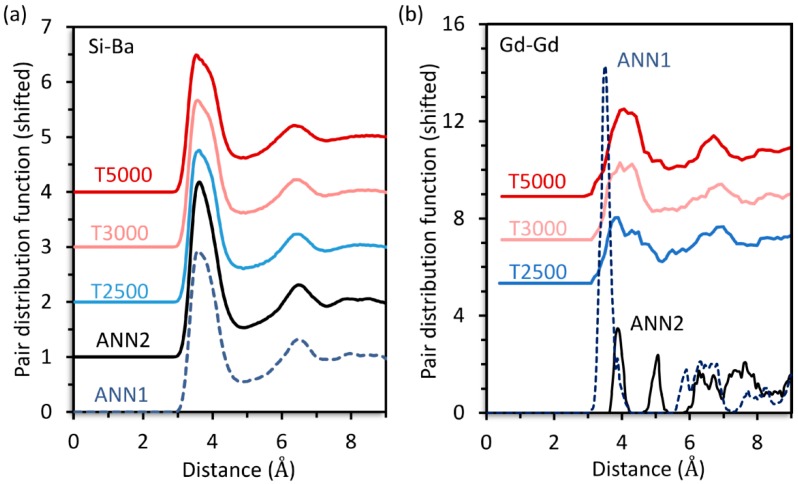
Pair distribution functions for **30Ba** of the Si-Ba (**a**) and Gd-Gd ion pairs (**b**) calculated by simulated annealing (ANN1, ANN2) and inherent structure sampling for different temperatures (T2500, T3000, T5000).

The Si-Ba, Figure 1a, and Gd-Gd, Figure 1b, pair distribution functions (PDF) for **30Ba** were calculated by simulated annealing (ANN), yielding the structure models ANN1 and ANN2, as well as inherent structure sampling (ISS) at different temperatures (T2500, T3000, T5000). For all predicted structures, the Si-Ba PDF are virtually identical, see Figure 1a. This also applies to every other PDF of the network former and modifier ions (not shown). However, the Gd-Gd PDF are very different for ANN1 and ANN2, see Figure 1b. By contrast, the ISS yields structures with similar Gd-Gd PDF that are nearly independent of the employed sampling temperature, see Figure 1b.

Table 2 summarizes the mass densities and relative potential energies Δ*E*_p_ with respect to the lowest energy structure (ANN1) for **30Ba** predicted by ANN and ISS. The calculated mass densities are 3.5 g/cm^3^ for ANN1 and ANN2, which is in good agreement with the experimentally observed value (3.54 g/cm^3^ [12]). In addition, the potential energies of both structures are almost the same. By contrast, the average mass densities and relative energies predicted by ISS increase with increasing sampling temperature ranging from 65 (T2500) to 119 meV/atom (T5000). Similarly, the mass densities are up to 0.2 g/cm^3^ (T5000) higher compared to ANN1 and ANN2. 

**Figure 2:**
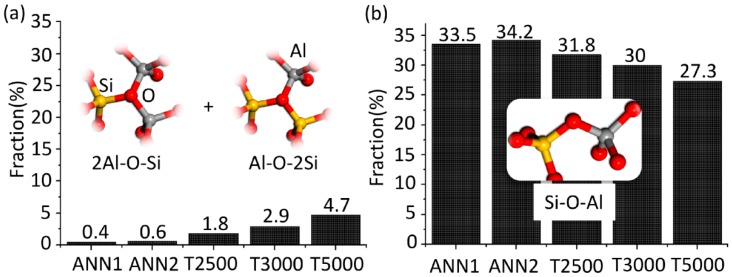
Fractions of triclusters of oxygen ions (**a**) and Al-O-Si bridging oxygen ions (**b**) for **30Ba** obtained by simulated annealing (ANN1, ANN2) and inherent structure sampling for different temperatures (T2500, T3000, T5000).

The most significant differences between the atomic structures obtained by ISS and ANN, which lead to higher densities and relative energies of the ISS predicted structures, are the fractions of triclusters (2Al-O-Si and Al-O-2Si) and bridging oxygen (Al-O-Si) ions bonded to [AlO*_x_*] or [SiO_4_], depicted in Figure 2. While almost none of such tricluster oxygen ions are present in ANN1 and ANN2, their fraction increases from 1.8 to 4.7% with increasing sampling temperature in the case of the ISS and is accompanied by a decrease of the Al-O-Si bridging oxygen fraction by approximately the same amount.

Both glass structure models predicted using the simulated annealing procedure (ANN1, ANN2) show virtually identical potential energies as well as short- and medium-range structures of network former and modifier ions. These findings demonstrate that the employed unit cell containing 6460 ions provides a sufficient sampling of atomic configurations to describe the glass network. However, including only 32 Gd^3+^ ions results in an inadequate sampling of the Gd^3+^ medium-range order, such as the Gd-Gd PDF, making precise modeling of the atomic structure impossible. Indeed, it was shown that structural models containing at least 80 rare earth ions are required [16] for reliable structure predictions of rare earth doped glasses using ANN. This means that in case of **30Ba**, a unit cell containing at least 16600 atoms is necessary to obtain a sufficient sampling of atomic configurations, which would result in computationally demanding simulations.

In contrast, the ISS uses unit cells containing only 2 Gd^3+^ and about 400 ions in total along with comparably short durations of MD simulations; hence considerably less computational effort is required. However, the sampling of the configuration space is not achieved by atomic structures within one single unit cell but by a large set of inherent structures (IS). Since the distribution *P*(*e*_IS_,*T*) of the IS energies *e*_IS_ corresponds to the probability that a configuration in the equilibrated liquid state is related to an IS with energy *e*_IS_ [21,22], it is possible to determine an average structure, see Equation (1), representing the liquid state at temperature *T*. The resulting medium-range structures of **30Ba**, i.e., the atomic structure starting from the second coordination shell of the ions such as the Si-Ba distribution, shows a very good agreement with the structures obtained by ANN. In addition, independent ISS at different temperatures yield very similar Gd-Gd PDF, showing that the sampling of the medium-range order of Gd^3+^ is sufficient to provide reproducible results.

However, structures predicted by ISS are slightly higher in energy and mass density due to the overestimation of the concentration of tricluster oxygen ions compared to ANN1 and ANN2. This is connected with the sampling of IS in the liquid state above *T*_g_. At higher sampling temperatures, i.e., higher kinetic energies of the ions, a larger number of IS of the PES are available lowering the probability of finding low energy structures. This leads to a shift of *P*(*e*_IS_,*T*) to higher *e*_IS_ with increasing sampling temperature [34] and, consequently, the predicted average IS show structural features such as tricluster oxygen ions that are higher in energy. In contrast, during the ANN such structural features are able to turn into lower energy structures (mainly bridging oxygen ions) until *T*_g_ is reached and the glass structure gets “frozen”. An additional equilibration performed for a geometrically optimized random structure (**30Ba**) for 1 ns at 2000 K showed no changes of the short- and medium-range structure. This means that *T*_g_, predicted by MD simulations along with the employed interatomic potential functions, is located between 2000 and 2500 K. An accurate calculation of thermodynamic quantities including *T*_g_ requires several ISS in this temperature range for evaluation of the temperature dependence of *P*(*e*_IS_,*T*) [21,34]. In contrast to the ISS, the ANN allows short-ranged relaxations between 2000 and 2500 K explaining the lower number of tricluster oxygen ions compared to the ISS. However, the slight deviations of tricluster oxygen fractions of less than 3% for T2500 and T3000 are expected to be smaller than the accuracy of structure predictions in general if empirically parameterized interatomic potential functions are applied.

One drawback of the ISS is that in the case of clustering of rare earth ions, the actual cluster structure comprising several Gd^3+^ ions cannot be predicted when only two Gd^3+^ ions are included in the structural model as in the present study. However, the employed ISS procedure allows estimation of the driving force for the formation of Gd-O-Gd contacts and, therefore, the determination of whether the tendency for Gd^3+^ clustering is preferred for the investigated glass compositions or not, see Section 3.3. Nevertheless, one advantage of the ISS, is the generation of a large set of structures which represent the liquid state close to *T*_g_, and show a close resemblance to the atomic configurations predicted by ANN but contain a considerably lower number of ions in the unit cells. This facilitates a simple yet efficient parallelization of computationally more demanding, quantum mechanical simulations. For instance, simulations at the density functional theory (DFT) level for unit cells containing several thousand atoms are tremendously challenging. In contrast, using at least a subset of atomic configurations obtained by ISS for structural optimizations at the DFT level would allow a refinement of the IS as well as *P*(*e*_IS_,*T*). Such a simulation approach could provide a deeper understanding of the relationship between the atomic structure and optical properties of rare earth doped aluminosilicate glasses.

### 3.2. Atomic Structure of Glass Network Formers and Modifiers Ions

The short-range order of the network builder ions is characterized by the first peak of the Si-O and Al-O pair distribution functions as well as the corresponding coordination numbers (not shown). The Si-O pair distribution functions of all glass compositions are as expected: The Si-O bond lengths show a narrow distribution and the mean bond length is 1.59 Å (1.60 Å in crystalline SiO_2_ [35], 1.61 Å in Mg_2_Al_3_[AlSi_5_O_18_] [36]). The Si-O coordination number is four. The Al-O bonds show a somewhat larger bond length of 1.71 Å in all cases (1.75 Å in Mg_2_Al_3_[AlSi_5_O_18_] [36]). The mean Al-O coordination number is 4.3 and 4.2 for **20Mg** and **20Ba**, respectively. Hence, the coordination number is a little bit higher than expected for an overall tetrahedral coordination. That means that small quantities of aluminum occur with a higher coordination number, i.e., with 5-fold (22.5% in **20Mg** and 16.7% in **20Ba**) or 6-fold (2.0% in **20Mg** and 1.2% in **20Ba**) coordination. Consequently, a corresponding small concentration of non-bridging oxygen should also occur. The Al-O coordination numbers for **30Mg** and **30Ba** are 4.2 and 4.1, respectively. They are somewhat smaller than in the metaluminous glasses due to the lower amount of 5- and 6-fold coordinated Al^3+^. The percentages are: 5-fold: 20.0% in **30Mg** and 9.4% in **30Ba** and 6-fold: 1.5% in **30Mg** and 0.4% in **30Ba**. 

Experimental studies were carried out using high-resolution magic angle spinning (HRMAS) NMR investigations on calcium aluminosilicate glasses with the same molar compositions of the undoped glass models (corresponding to **20Ca** and **30Ca**, cf. Table 1). It was observed that the concentrations of 5-fold coordinated Al^3+^ are about 3% (**20Ca**) [37] and 5% (**30Ca**), respectively [38]. In addition, it was found that alkaline earth ions with higher field strengths (Mg^2+^ > Ca^2+^ > Ba^2+^) favor the formation of 5-fold and 6-fold coordinated Al^3+^, while the concentration of Al^3+^ in 6-fold coordination is considerably lower than that in 5-fold coordination [39]. These observations are qualitatively in agreement with the simulation results, yet the absolute value of 5-fold coordinated Al^3+^ is clearly overestimated. This is possibly connected with the inaccuracy of the empirical parameterization employed for the Al-O interatomic potential functions. However, the employed simulation procedure facilitates the refinement of the obtained structure models using higher-level simulations, e.g., at the DFT level, see Section 3.1.

Table 3 shows the distances and coordination numbers (CN) of the first and second coordination sphere of the network-modifier (NM) ions. In the case of **20Mg** and **20Ba,** the calculated Mg-O and Ba-O bond lengths are 2.01 and 2.79 Å and the CN 4.6 and 9.1, respectively. Of course, the larger ionic radius of the Ba^2+^ ions should result in longer bonds and a higher CN than for the smaller Mg^2+^. Since the [AlO_4_]^−^ tetrahedra need a cation for charge compensation, the distances Mg-Al and Ba-Al are also of interest. They are 3.27 and 3.57 Å, while the calculated CN are 2.9 and 4.3 for Al-Mg and Al-Ba, respectively. The bond lengths Mg–O and Ba–O for **30Mg** and **30Ba** are 2.01 Å and 2.79 Å and hence identical with those obtained for **20Mg** and **20Ba**. The calculated CN are 4.7 and 8.3, respectively. Hence, the NM–O CN for **30Mg** is significantly smaller than for **30Ba**.

The CN of NM ions shown in Table 3 for the second coordination sphere, i.e., their coordination with other cations, is substantially different for the peralkaline glass structure models (**30Mg**, **30Ba**) compared to the metaluminous compositions (**20Mg**, **20Ba**). As expected from the chemical compositions, the coordination of NM ions with [AlO*_x_*] polyhedra is more likely in the metaluminous glasses than in peralkaline compositions, while in the peralkaline compositions the coordination with other NM ions is more likely. This also shows that the so-called depolymerized regions in the glass structure must be larger in the peralkaline glasses. However, the most numerous coordination partners of the NM ions are [SiO_4_] tetrahedra. Interestingly, the number of coordinating [SiO_4_] tetrahedra is nearly constant for each of the network-modifying atoms (Mg^2+^, Ba^2+^) in peralkaline and metaluminous glasses. That means their coordination with [SiO_4_] tetrahedra, similarly to their coordination with oxygen, depends only on the radius of the network-modifying ion.

In Table 4 the oxygen coordination numbers of the NM ions are separated into coordination with bridging oxygen (BO), i.e., ≡(Si, Al)−O−(Si, Al)≡, and non-bridging oxygen (NBO), i.e., ≡Si−O, as well as oxygen ions in triclusters (Tri). As can be seen from the data, the coordination with NBO is generally higher for the peralkaline compositions **30Mg** and **30Ba**. As explained in the introduction section, this is due to the higher NMO/Al_2_O_3_ ratio in these glasses. Interestingly, the data shows that a notably higher number of oxygen ions are of the non-bridging species in the metaluminous magnesium containing **20Mg** (31.1%) compared to its barium containing counterpart **20Ba** (16.5%). Due to the smaller size of the Mg^2+^ ion and the repulsion between two [AlO_4_]^−^ tetrahedra the charge compensation of two [AlO_4_]^−^ tetrahedra by only one NM^2+^ is less likely in metaluminous magnesium aluminosilicate glasses than in barium aluminosilicate glasses and NBO sites are formed instead [40,41]. In a macroscopic scale, this effect results in a lower glass transition temperature, *T*_g_, and lower melt viscosity of the **20Mg** composition in comparison to the **20Ba** composition, while exactly the opposite effect occurs for the peralkaline compositions. Here, **30Ba** has a lower glass transition temperature and lower viscosity than **30Mg** [14].

**Figure 3:**
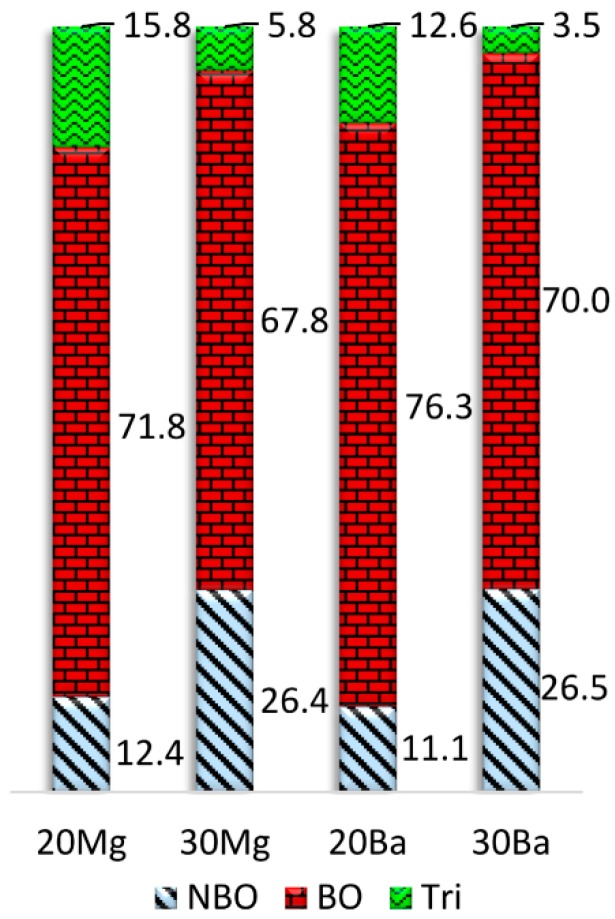
Fractions of non-bridging oxygen (NBO), bridging oxygen (BO), and tricluster oxygen ions (Tri) as a function of chemical composition.

A comparison of the average bridging oxygen (BO), NBO, and oxygen tricluster fractions calculated for all oxygen ions of the glass structure models is depicted in Figure 3. While the number of bridging oxygen slightly increases, a considerable number of NBO turn into oxygen triclusters when the concentration of NM ions is increased. However, it must be noted that the number of oxygen triclusters is slightly overestimated by the employed structure prediction procedure. The higher number of NBO sites in **20Mg** compared to **20Ba** additionally results in a higher number of oxygen triclusters in this composition, since the triclusters ensure charge compensation of the remaining [AlO_4_]^−^ tetrahedra. In addition, the number of triclusters is drastically reduced in the peralkaline compositions **30Mg** and **30Ba** compared to **20Mg** and **20Ba** due to their higher NMO/Al_2_O_3_ ratio. Accordingly, the coordination of the NM ions with oxygen triclusters is notably smaller for the peralkaline glasses **30Mg** and **30Ba**, see Table 4. In the case of calcium aluminosilicate glasses (**20Ca**, **30Ca**), HRMAS NMR investigations found concentrations of about 4% NBO for **20Ca** [37] and 23% NBO for **30Ca** [38]. Furthermore, it was observed that the NBO content is virtually independent of the used alkaline earth oxide [39]. Except for the overestimation of the NBO fraction for the metaluminous glass composition (**20Mg**, **20Ba**), the simulation results are in good agreement with these experimental observations. As mentioned above, a further refinement of the structural models can be achieved by employing higher-level simulations, see Section 3.1.

### 3.3. Medium-Range Structure of Gd^3+^

**Figure 4:**
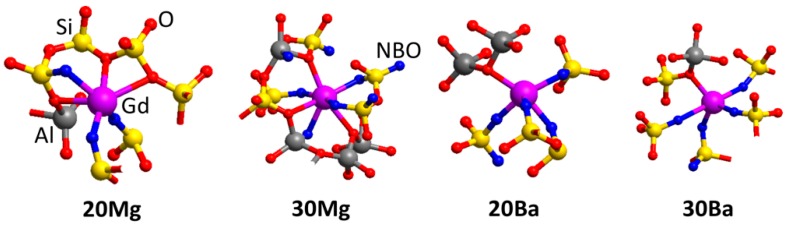
Examples for structural models of the first and second coordination shell of Gd^3+^ ions. Non-bridging oxygens (NBO) are highlighted in blue.

Table 5 summarizes the coordination numbers (CN) of Gd^3+^ ions for all glass structural models. Examples of the atomic structure for the first and second coordination shell of Gd^3+^ of the glass models are depicted in Figure 4. The coordination of Gd^3+^ with [AlO*_x_*] and [SiO_4_] shows virtually the same dependence on the chemical composition as the NM ions. While the number of [SiO_4_] units in the second coordination shell is approximately constant, the coordination with [AlO*_x_*] polyhedra increases with increasing Al_2_O_3_ concentration.

In contrast, significant changes are found for the coordination of Gd^3+^ ions to the NM ions, which is steadily increasing in the order **20Mg** < **20Ba** < **30Mg** < **30Ba**. For the peralkaline composition **30Ba**, Gd^3+^ ions have by far the highest coordination number to other network modifier (NM) ions (Ba in this case). In fact, this composition is the only the case where the Gd^3+^ coordination is notably different from all other glass structural models. The same applies to the Ba^2+^ ions showing a considerably higher NM-NM coordination compared to the other glass structure models, see Table 3. This clearly shows that Gd^3+^ is incorporated into the glass structure in the same way as a network-modifying ion. However, despite its increasing coordination to NM ions and increasing Gd-NM distances depending on the glass composition, the distance to directly neighboring oxygen atoms is constant at 2.25 Å for all four glass structure models. Also, the number of neighboring oxygen ions is mostly independent of any change in the glass composition. It varies only between 5.4 and 5.8 in all four predicted glass structures.

The Gd-Gd CNs shown in Table 5 are lower than 0.1 for all structural models showing that, on average, less than 10% of the Gd^3+^ ions of the IS form Gd-O-Gd contacts. This indicates a low driving force for clustering of Gd^3+^ ions in the investigated aluminosilicate glasses. Therefore, the formation of larger Gd*_x_*O*_y_* clusters appears to be unlikely. In addition, the Gd-Gd CN is almost independent of the chemical composition since such small changes are expected to be lower than the accuracy of present structural predictions using empirically parameterized interatomic potential functions.

Table 6 summarizes the coordination numbers of Gd^3+^ with bridging (BO), non-bridging (NBO), and tricluster (Tri) oxygen ions. The coordination of Gd^3+^ with NBO is somewhat higher in the barium-containing glass compositions than in the respective magnesium-containing glass structure models. Obviously, ions of higher electric field strength, i.e., ions with a smaller ionic radius or higher charge, such as Mg^2+^ and Gd^3+^ prefer coordination with NBO sites. In addition, in analogy to the NM ions, the coordination of Gd^3+^ with oxygen ions in triclusters is lower in the peralkaline compositions (**30Mg**, **30Ba**) compared to the metaluminous compositions (**20Mg**, **20Ba**), which correlates with the total concentration of tricluster oxygen ions, see Figure 3. 

Table 7 shows the coordination numbers of Gd^3+^ ions with BO and NBO separated according to the network former ions present in the second coordination sphere. It can be differentiated between Si-bound (^−^O−Si≡) and Al-bound (^−^O−Al≡) NBO and BO, i.e., ≡(Si, Al)−O−(Si, Al)≡, or BO in triclusters. The overall coordination of Gd^3+^ with NBO increases with the increasing radius and concentration of the network-modifying ions, see Table 6. However, as shown in Table 7, this increase is only caused by additional Si-bound oxygen. The percentage of Al-bound non-bridging oxygen is virtually constant for all glasses. The increase in the numbers of Gd-coordinated NBO is realized at the expense of the numbers of BO. But here the number of ≡Si−O−Si≡ species remains almost unchanged, while the number of ≡Al−O−Al≡ species is the most diminished. In summary, it can be stated that the results show a clear tendency for an increased number of Si-bound NBO in the coordination sphere of the doped Gd^3+^ ions with increasing size and concentration of the network-modifying ions, while the absolute number of coordinating Si^4+^ in the second coordination sphere remains approximately constant, as stated earlier, see Table 5. Additionally, the Al^3+^ ions in the second coordination sphere with their high field strength are replaced by the relatively low field strength network-modifier ions Mg^2+^ and Ba^2+^. This replacement is more apparent in the order **20Mg** < **20Ba** < **30Mg** < **30Ba**. A calculation of the summed-up field strengths at the Gd^3+^ positions resulting from the coordinating cations Si^4+^, Al^3+^, Mg^2+^, and Ba^2+^, according to the data in Table 5, gives an order **20Mg** ≈ **30Mg** > **20Ba** > **30Ba**. This very simplified model can be applied since the number and distance of the directly neighboring oxygen atoms is mostly independent of the glass composition, see Table 5. Despite different network-modifier CN and distances, the field strengths for **20Mg** and **30Mg** are almost the same. This result correlates perfectly with the peak splitting in Tb^3+^ doped glasses [14]: Higher concentrations of low field strength ions in the glass composition increase the peak splitting and therefore induce an increased electric field at the rare earth positions; most likely due to their lower polarization power on the neighboring oxygen atoms. On the other hand, these results do not correlate with the experimentally derived luminescence lifetime data: The lifetimes for all tested rare earth ions Sm^3+^, Eu^3+^, and Tb^3+^ increase in the order **20Ba** < **20Mg** = **30Mg** < **30Ba** [11,12,14]. The respective lifetimes for the samples **20Mg** and **30Mg** are almost the same. However, it must be stated that in the cases of Sm^3+^ and Tb^3+^, lifetimes from **30Ba** compositions were not reported. Instead, the results for the very similar composition with 35 mol% BaO, 10 mol% Al_2_O_3_, and 55 mol% SiO_2_ have been used for these comparisons. In summary, the structural models of the glasses presented here are not sufficient to explain all the observed luminescence effects. However, a deeper understanding of the relation between the local structure of rare earth ions in aluminosilicate glasses and the optical properties can possibly be achieved using higher level quantum mechanical simulations employing the provided structural models.

## 4. Conclusions

In summary, structural predictions of Gd_2_O_3_ doped aluminosilicate glasses containing BaO and MgO are performed for investigation of the atomic medium-range structure of Gd^3+^ as a function of a network-modifier oxide to Al_2_O_3_ ratio varied between 1 and 3. The simulation procedure used, denoted here as inherent structure sampling (ISS), samples the potential energy surface at temperatures slightly above the glass transitions temperature *T*_g_, assuming that the resulting averaged structures resemble the atomic configurations below *T*_g_. This allows the statistical sampling of the medium-range structures of Gd^3+^ using unit cells containing a considerably smaller number of ions compared to standard simulated annealing procedures. The slight deviations of the short-range structure of network former and modifier ions predicted by ISS from the results of the simulated annealing procedure are expected to be lower than the accuracy of structural predictions in general if empirically parameterized interatomic potential functions are applied. 

It can be stated that there is a clear tendency for increased numbers of Si-bound non-bridging oxygen atoms in the coordination sphere of the doped Gd^3+^ ions with increasing ionic radius and concentration of the network-modifying ions at constant SiO_2_ concentrations in the glass composition. In addition, a low tendency for the formation of Gd–O–Gd contacts is found. However, the actual relationship between the atomic structure and experimentally observed luminescence properties cannot be conclusively clarified. However, the provided structural models can be used as a starting point for more accurate quantum mechanical simulations.

It was also shown that different network-modifying ions are incorporated into the glass structure in different ways depending on their radius and charge. Larger ions have higher coordination numbers and higher distances to their neighboring atoms. However, smaller and higher charged network-modifiers, i.e., high electric field strength ions, prefer coordination with non-bridging oxygen sites, while low field strength ions prefer coordination with bridging oxygen. In other words, high field strength network-modifying ions compete with other high field strength ions, such as, e.g., Gd^3+^ in their coordination with non-bridging oxygen sites, and therefore diminish the non-bridging oxygen coordination of Gd^3+^. Additionally, they actually decrease the electric field strength at the rare earth positions. This effect could be exploited to tailor the optical properties of the doped ions and to develop new optically active materials.

## Figures and Tables

**Table 1:** Unit cell compositions of the glass structure models.

		Chemical Composition [mol%]				
Model	Unit Cell	BaO	MgO	Al_2_O_3_	SiO_2_	Gd_2_O_3_
**20Mg**	Gd_2_Mg_25_Al_50_Si_75_O_253_	-	19.8	19.8	59.6	0.8
**30Mg**	Gd_2_Mg_42_Al_28_Si_83_O_253_	-	30.0	10.0	59.3	0.7
**20Ba**	Gd_2_Ba_25_Al_50_Si_75_O_253_	19.8	-	19.8	59.6	0.8
**30Ba**	Gd_2_Ba_42_Al_28_Si_83_O_253_	30.0	-	10.0	59.3	0.7

**Table 2:** Mass density *ρ* and relative energies Δ*E*_p_ with respect to the lowest energy structure for **30Ba** calculated by simulated annealing (ANN1, ANN2) and inherent structure sampling at different sampling temperatures (T2500, T3000, T5000).

Model	*ρ* (g/cm^3^)	Δ*E*_p_ (meV/atom)
ANN1	3.50	0
ANN2	3.50	2
T2500	3.55	65
T3000	3.59	83
T5000	3.70	119

**Table 3:** Coordination numbers CN (and interatomic distances [Å]) of the network-modifier (NM) ions obtained for different chemical compositions.

CN (Distance)	20Mg	30Mg	20Ba	30Ba
NM-O	4.5	4.7	9.1	8.3
	(2.01)	(2.01)	(2.79)	(2.79)
NM-Al	2.9	1.6	4.3	2.1
	(3.27)	(3.21)	(3.57)	(3.57)
NM-Si	4.1	4.5	7.3	7.0
	(3.21)	(3.27)	(3.63)	(3.63)
NM-NM	1.6	2.7	2.9	5.1
	(2.91)	(2.97)	(4.11)	(4.17)

**Table 4:** Separation of NM-O coordination numbers (CN) of network-modifier ions (NM) into nonbridging oxygen (NBO), bridging oxygen (BO), and tricluster oxygen ions (Tri). Fractions [%] are given in brackets.

CN (Fraction)	20Mg	30Mg	20Ba	30Ba
NM-NBO	1.4	2.3	1.5	2.7
	(31.1)	(48.9)	(16.5)	(32.5)
NM-BO	3.0	2.4	7.1	5.5
	(66.7)	(51.1)	(78.0)	(66.3)
NM-Tri	0.1	0.0	0.5	0.1
	(2.2)	(0.0)	(5.5)	(1.2)

**Table 5:** Coordination numbers CN (and interatomic distances [Å]) of Gd^3+^ ions obtained for different chemical compositions.

CN (Distance)	20Mg	30Mg	20Ba	30Ba
Gd-O	5.8	5.6	5.4	5.5
	(2.25)	(2.25)	(2.25)	(2.25)
Gd-Al	3.2	1.8	2.9	1.4
	(3.51)	(3.63)	(3.57)	(3.51)
Gd-Si	4.7	5.3	4.5	4.5
	(3.57)	(3.57)	(3.57)	(3.51)
Gd-NM	2.2	3.3	3.1	4.4
	(3.27)	(3.33)	(3.99)	(3.93)
Gd-Gd	0.07	0.09	0.08	0.06
	(3.60)	(3.60)	(3.60)	(3.60)

**Table 6:** Separation of Gd-O coordination numbers (CN) into nonbridging oxygen (NBO), bridging oxygen (BO), and tricluster oxygen ions (Tri). Fractions [%] are given in brackets.

CN (Fraction)	20Mg	30Mg	20Ba	30Ba
Gd-NBO	2.8	3.8	3.0	4.2
	(48.3)	(67.9)	(55.6)	(76.4)
Gd-BO	2.9	1.8	2.3	1.3
	(50.0)	(32.1)	(42.6)	(23.6)
Gd-Tri	0.1	0.0	0.1	0.0
	(1.7)	(0.0)	(1.8)	(0.0)

**Table 7:** Fractions [%] of bridging (BO) and non-bridging oxygen (NBO) atoms within the first coordination shell of Gd^3+^ separated according to the second-nearest neighbors.

Gd^3+^ Coordination		20Mg	30Mg	20Ba	30Ba
NBO	Gd-O-Si	38.2	57.3	45.7	67.2
	Gd-O-Al	10.7	9.9	10.7	9.9
BO	Gd-O-2Al	10.5	3.9	11	3.5
	Gd-O-2Si	9.9	11.1	6.8	6.5
	Gd-O-SiAl	29.2	17.4	24.7	12.8
	Gd-Tri	1.5	0.4	1.1	0.1
